# Myostatin Suppression of Akirin1 Mediates Glucocorticoid-Induced Satellite Cell Dysfunction

**DOI:** 10.1371/journal.pone.0058554

**Published:** 2013-03-13

**Authors:** Yanjun Dong, Jenny S. Pan, Liping Zhang

**Affiliations:** Department of Medicine, Nephrology Division, Baylor College of Medicine, Houston, Texas, United States of America; The Chinese University of Hong Kong, China

## Abstract

Glucocorticoids production is increased in many pathological conditions that are associated with muscle loss, but their role in causing muscle wasting is not fully understood. We have demonstrated a new mechanism of glucocorticoid-induced muscle atrophy: Dexamethasone (Dex) suppresses satellite cell function contributing to the development of muscle atrophy. Specifically, we found that Dex decreases satellite cell proliferation and differentiation *in vitro* and *in vivo*. The mechanism involved Dex-induced upregulation of myostatin and suppression of Akirin1, a promyogenic gene. When myostatin was inhibited in Dex-treated mice, Akirin1 expression increased as did satellite cell activity, muscle regeneration and muscle growth. In addition, silencing myostatin in myoblasts or satellite cells prevented Dex from suppressing Akirin1 expression and cellular proliferation and differentiation. Finally, overexpression of Akirin1 in myoblasts increased their expression of MyoD and myogenin and improved cellular proliferation and differentiation, theses improvements were no longer suppressed by Dex. We conclude that glucocorticoids stimulate myostatin which inhibits Akirin1 expression and the reparative functions of satellite cells. These responses attribute to muscle atrophy. Thus, inhibition of myostatin or increasing Akirin1 expression could lead to therapeutic strategies for improving satellite cell activation and enhancing muscle growth in diseases associated with increased glucocorticoid production.

## Introduction

Satellite cells are mononucleated muscle precursor cells which play critical roles in the growth, maintenance and repair of skeletal muscle [1]. In mouse models of muscle atrophy (i.e., aging, myopathy, or muscle denervation) the number and activity of satellite cells are reduced suggesting that satellite cell dysfunction contributes to the development of muscle atrophy [2], [3]. In a mouse model of chronic kidney disease (CKD), we found impaired satellite cell proliferation and differentiation which delayed muscle regeneration and decreased muscle mass [4]. These reports suggest that functional satellite cells are required to maintain muscle mass in catabolic conditions.

Catabolic conditions are often associated with elevated glucocorticoid (GC) production [5], [6]. This is relevant because an excess of GC can contribute to muscle wasting [5]–[7]. Whether GCs contribute to muscle mass loss by impairing satellite cell function is unknown. But, it is reported that dexamethasone, a potent synthetic member of the glucocorticoid class of steroid drugs, inhibits myoblast proliferation and differentiation and decreases myogenic gene expression [8]. However, the signaling pathways by which GCs lead to decreased myogenic gene expression are not clear. One possibility is that GCs increase myostatin secretion [9], [10]. This is relevant because myostatin expression is increased in muscles of rodents developing muscle atrophy and can negatively regulate satellite cell activation and self-renewal [11]–[14]. In the current study, we tested the hypothesis that GCs cause muscle loss by triggering myostatin expression with impaired satellite cell function. The mechanism by which myostatin suppresses satellite cell activation is unknown but one candidate is activation of Smad3 signaling [15], [16]. Another candidate pathway involves Akirin1, because suppressive subtraction hybridization analyses identified that Akirin1 is specifically regulated by myostatin [17].

Akirins are 20–25 kDa nuclear proteins that regulate gene expression in processes such as innate immune response or carcinogenesis [18]. They interact with cofactors to either promote or repress mRNA transcription including 14-3-3 proteins [18] or the basic helix-loop-helix transcription factor, Twist [19]. In the present studies, we found that GCs suppress satellite cell activation *in vivo* and *in vitro* and we uncovered that the mechanism involves an upregulation of myostatin and downregulation of Akirin1 which leads to inhibition of MyoD.

## Materials and Methods

### Reagents and Antibodies

Antibodies against p-Akt (Ser473) (Cell Signaling Technology, Beverly, MA), the glucocorticoid receptor (GR) (Santa Cruz Biotechnology, Santa Cruz, CA), MyoD (Vector Laboratories, Burlingame, CA), eMyHC and myogenin (Developmental Studies Hybridoma Bank, University of Iowa, Iowa City, IA), Akirin1 (Sigma-Aldrich, St. Louis, MO), myostatin (Abcam, Cambridge, MA), and Atrogin-1 and MuRF-1 (ECM Bioscience; Versailles, KY) were used according to the companies’ protocols. The anti-Ki67-Alexa Fluor® 555 antibody was from BD Bioscience (San Jose, CA). Cardiotoxin (CTX) and dexamethasone (Dex) were from Sigma-Aldrich and the anti-myostatin peptibody (myostatin inhibitor) was from Amgen (Amgen, Thousand Oaks, CA, USA) [20].

### Animal Experiments

All animal experiments and procedures were approved by the Baylor College of Medicine Institutional Animal Care and Use Committee. C57/BL6 mice (Jackson Lab, Bar Harbor, ME, 10 to 12 weeks) were intraperitoneally injected with Dex (5 mg/kg) twice a day for 14 days [21], [22]. The anti-myostatin peptibody (5 mg/kg, IC50 ∼1.2 nM) [20] was injected subcutaneously every other day into one of a pair of Dex-injected mice; the other Dex-treated mice was injected with an equal volume of PBS (Control). The mice were pair-fed and body weights were assessed daily. Gastrocnemius, tibialis anterior (TA), soleus and extensor digitorum longus (EDL) muscles were removed and weighed at the time of mice sacrifice.

Transgenic mice bearing MyoD-Cre were a gift from Dr David J. Goldhamer (U. Connecticut, Storrs, CT) [23]. MyoD-Cre mice were crossed with floxed-GR transgenic mice [22] to create glucocorticoid receptor knockout mice (GRKO).

### Muscle Injury and Regeneration Model

Normally satellite cells are quiescent, but they are activated by stress (including weight bearing, trauma or injury) to proliferate, differentiate and fuse into multinucleated myofibers [24]. We injured TA muscle with cardiotoxin (CTX) in order to study satellite cell function in muscle [4], [25], [26]. For example, one TA muscle of each mouse was injected with 80 µl of 10 µM CTX; the contralateral TA muscle was injected with an equal volume of PBS (control). Mice were sacrificed at different times after injury and both TA muscles were collected and either embedded with a medium consisting of polyethylene glycol and polyvinyl alcohol and frozen in dry-ice chilled isopentane for histological analysis or muscles were stored in liquid nitrogen until protein or RNA was isolated. Myogenic markers of satellite cells in injured muscles were examined by immunostaining, RT-PCR or western blotting.

### Satellite Cell Isolation, Proliferation and Differentiation Assay

Satellite cell isolation and identification were performed as described [4]. Briefly, skeletal muscles were digested with 0.2% Collagenase Type II (Worthington Biochemical, Lakewood, NJ) in DMEM plus 1% penicillin/streptomycin at 37°C for 30 minutes. The mixture was passed through a 100 µm filter and then subjected to Percoll gradient centrifugation. Satellite cells were obtained from the interface between 40% and 70% of Percoll and immunostained with anti-Pax-7; >95% of cells were Pax-7 positive [4]. RNA and protein were isolated from satellite cells or the cells were cultured on Matrigel-coated plates (BD Bioscience, San Jose, CA) in growth media (DMEM with 20% FBS, 1% penicillin/streptomycin, 40 µg/ml gentamicin and 2% chicken embryo extract). Satellite cell proliferation was assessed by co-immunostaining of anti-Pax-7 with anti-Ki67. The average percentage of the number of Pax7 and Ki67 dual positive cells to total Pax7 positive cells in 10 areas was designated as the proliferation rate.

Some of the satellite cells were converted into myotubes by incubation in differentiation media (DMEM plus 2% horse serum, 1% penicillin/streptomycin) for 4 days. Myotubes were fixed in 2% paraformaldehyde for 10 min before they were immunostained for anti-eMyHC. The differentiation index was calculated as the sum of nuclei within myotubes that stained positively for eMyHC plus the number of eMyHC positive, mononucleated cells divided by the total number of nuclei [27].

Satellite cells also were transducted with shRNA-myostatin or shRNA-control lentivirus. The shRNA-myostatin lentiviral particles contain 3 target-specific constructs that encode 19–25 nt shRNA designed to knock down myostatin expression; shRNA-control lentivirus contain a shRNA construct encoding a scrambled sequence that will not lead to the specific degradation of any known cellular mRNA. Lentiviral particles of shRNA-myostatin or shRNA-control were from Santa Cruz Technology. Freshly isolated satellite cells were plated into 12 wells plates and incubated in growth media for 3 days to allow cell attachment, then satellite cells were transducted with 20 µl of 1×10^6^ infectious units of virus (IFU) of shRNA-myostatin or shRNA-control with 8 µg/ml polybrane. These cells were then treated with Dex or recombinant myostatin and co-immunostained with Pax7 and Ki67 to evaluate satellite cell proliferation. Other cells transducted with shRNA lentivirus were differentiated into myotubes and immunostained with eMyHC to evaluate satellite cell differentiation in the presence of GC or myostatin.

### RT-PCR Analysis

RT-PCR was performed as described [4], [28] with relative gene expression calculated from cycle threshold (Ct) values using GAPDH or 18S as an internal control (relative expression = 2^(sample Ct − GAPDH Ct)^). Primers and their sequences are listed in Table 1.

**Table 1:** RT-PCR primer sequences.

mRNA	Forward	Reverse
Myf5	5′-ATCTGGCTTCTCTCTCTCCAGTTG-3′	5′-TTAGGCCCTCCTGGAAGAAGTCAT-3′
Akirin1	5′-GCAGAACAGTATGAATCG-3′	5′-AGGCTTCAGGATACATAG-3′
Myostatin	5′-TGGCATTACTCAAAAGCAAAAAG-3′	5′-CATCAATACTCTGCCAAATACCA-3′
Myogenin	5′-ACAGCATCACGGTGGAGGATATGT-3′	5′-CCCTGCTACAGAAGTGATGGCTTT-3′
MyoD	5′-ACGACTGCTTTCTTCACCACTCCT-3′	5′-TCGTCTTAACTTTCTGCCACTCCG-3′
GAPDH	5′-ACCACCATGGAGAAGGCCGG-3′	5′-CTCAGTGTAGCCCAAGATGC-3′
18S	5′-GAAACGGCTACCACATCCAAGG-3′	5′-GTCCCTCTTAATCATGGCCTCAG-3′

### Immunohistochemical Analyses

Serial, transverse cryo-sections (8 µm thick) of TA muscles were air-dried and fixed in cold acetone or 4% paraformaldehyde for 10 min. before staining with hematoxylin and eosin (H&E). Tissue sections were immunostained for eMyHC to evaluate new-myofiber formation. To calculate the cross-sectional areas of individual myofibers, TA muscles were first immunostained with anti-laminin to define the basement membrane. Fiber sizes were measured using Nikon NIS-Elements Br 3.0 software (Melville, NY). The distribution of myofibers was expressed as the percentage of myofibers within a designated size range divided by the total number of myofibers. The measurement was made by observers masked to the treatment.

### Cell Transfection

C2C12 myoblasts were transfected with plasmids expressing Akirin1 or cDNA3 using Amaxa Nucleofector technology (Lonza, Allendale, NJ). Cells were collected by centrifugation at 450 g for 5 minutes at room temperature and 1×10^6^ cells were re-suspended in 100 µl of Basic Nucleofector reagent. The cell suspension mixed with 2 µg plasmid was transfected by electroporation. Transfection success was >90% with this method. The electro-transfected cells were transferred to a 6 well plate and allowed to grow or differentiate. Following treatment with Dex (10 µM), the proliferation or differentiation rates were evaluated from Ki67 or eMyHC immunostaining, respectively.

### Establishing Stable Clones with shRNA-myostatin

shRNA-myostatin and shRNA-control lentiviral particles were bought from Santa Cruz Technology. C2C12 myoblasts were grown in a 12-well plate with DMEM plus 10% FBS for 24 h before viral infection (approximately 50% confluent on the day of infection). The cell culture media was changed to new DMEM plus 10% FBS, 8 µg/ml polybrene and 1×10^6^ infectious units of virus. After 24 h, the media was changed to DMEM plus 10% FBS for another 24 h. The cells were split in a 1 to 5 concentration and replated in different concentrations of puromycin (2, 5 or 10 µg/ml); 5 ug/ml puromycin achieved the best selection. The selected clone by knocked down of myostatin was evaluated by western blot and RT-PCR.

### Statistical Analysis

Values are presented as mean ± SEM. Data were compared across different treatment and time points using two-way ANOVA. Student’s ***t***-test was performed with statistical significance (p<0.05). A minimum of three replicates were performed for each experimental condition.

## Results

### Dex Suppresses Satellite Cell Function in vitro

**Figure 1:**
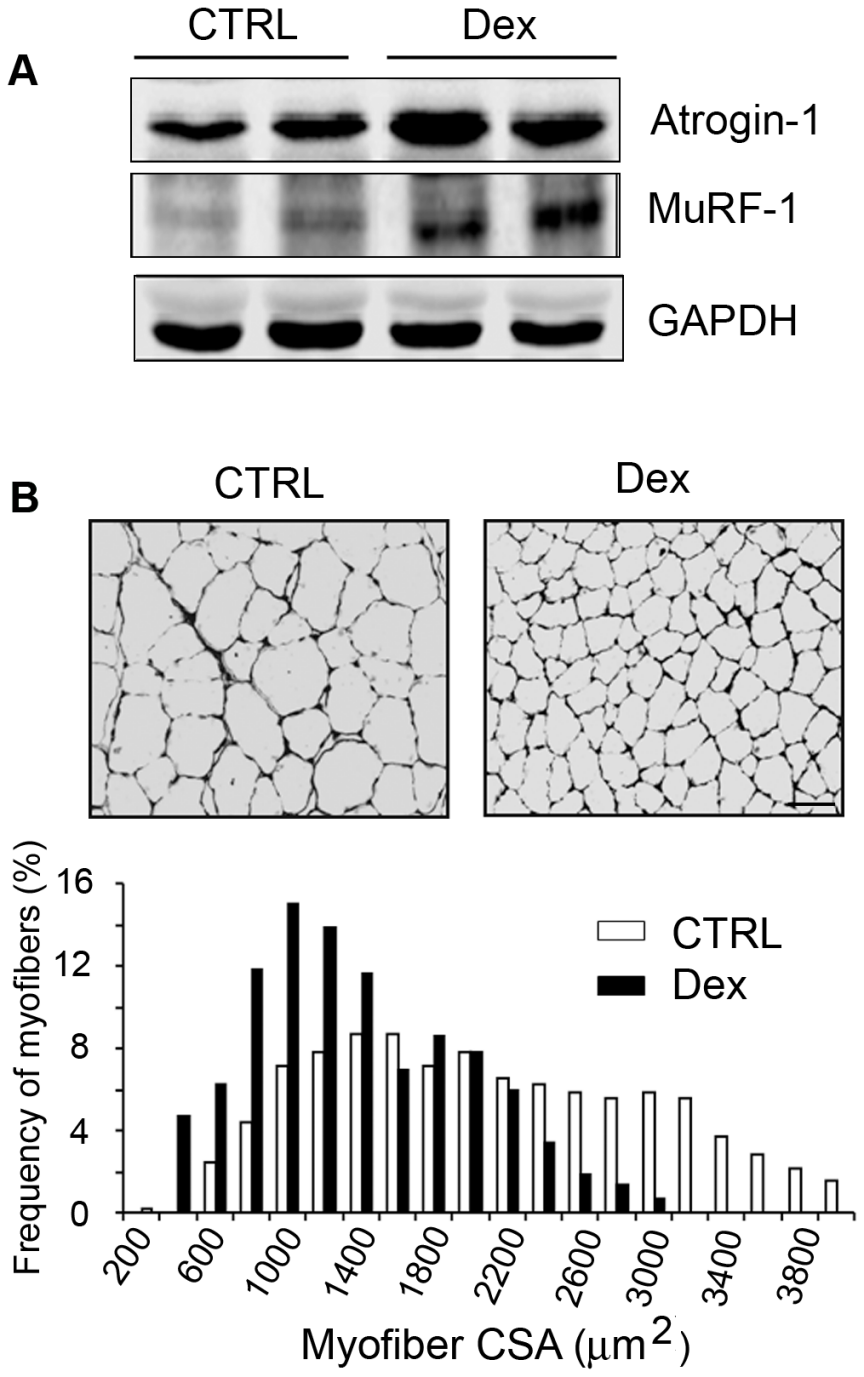
Dex induces muscle proteolysis through the ubiquitin-proteasome system. C57/BL6 mice were treated with Dex for 14 days. A. A representative western blot of Atrogin-1 or MuRF-1 in muscles. B. Cross-sections of TA muscles were immunostained with anti-laminin (upper panel, bar = 50 µm). Myofiber areas were measured and their distribution was calculated as the percentage of the number of myofibers in a designated area divided by the total number of myofibers assessed (lower panel).

Consistent with others, we found that high doses of Dex (5 mg/kg) induced a significant decrease in body and muscle weights *vs.* results in untreated, control mice (Table 2) [29]–[31]. There also was activation of the ubiquitin-proteasome system as evidenced by increased expression of E3 ubiquitin ligases, Atrogin-1 or MuRF-1 in muscles (Fig. 1A). Dex-induced loss of muscle weight was further demonstrated by the finding that the distribution of myofibers in TA muscles of Dex-treated mice was shifted to the left (smaller direction) (Fig. 1B).

**Table 2:** Dex treatemnt decreases body and muscle weight in mice.

	BW (g)	Gastr. (mg)	TA(mg)	Soleus(mg)	EDL(mg)
Ctrl	26±0.56	132.66±1.48	47.23±0.98	9.14±0.47	9.32±0.35
Dex	22.3±0.86	113.66±1.79	40±0.59	7.34±0.90	8.17±0.45
t-test	0.016	0.017	0.006	0.132	0.009

12 week-old male mice (C57/BL6) were injected with Dex (5 mg/kg twice a day) for 14 days, n = 5 mice in each groups.

**Figure 2:**
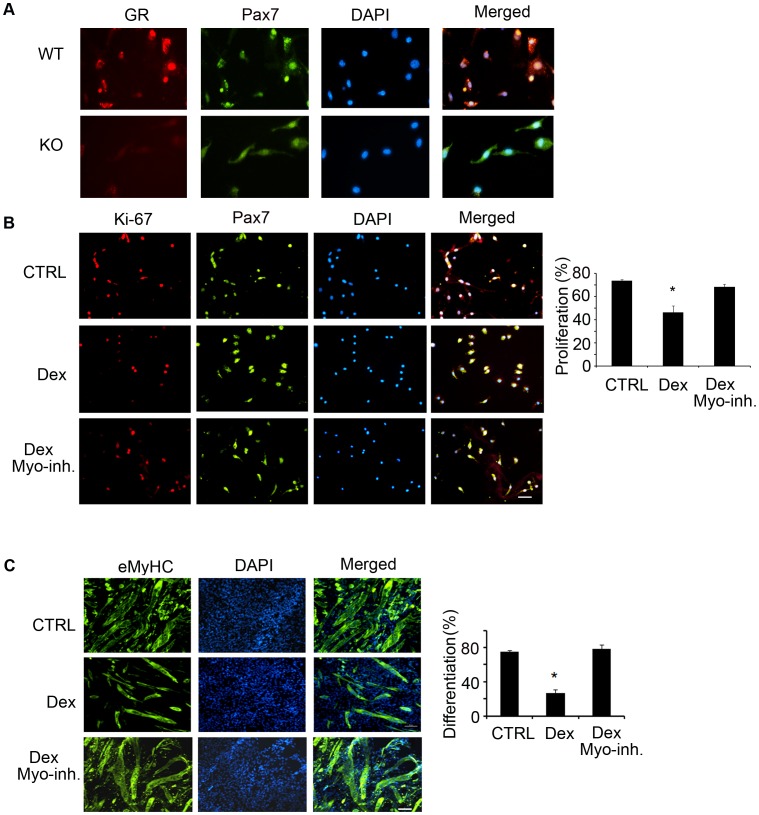
Dex impairs satellite cell function *in vitro*. A. Satellite cells were co-immunostained with anti-GR (Red) or Pax7 (green); nuclei were stained with DAPI (blue). In the merged picture (right column), satellite cells expressing glucocorticoid receptor (GR) are yellow (bar = 50 µm). B. Satellite cells were treated with Dex plus PBS or Dex plus myostatin inhibitor for 24 h, the percentage of Ki67 and Pax7 dual-positive (yellow) cells to total Pax-7 positive cells was calculated as the satellite cell proliferation rate (right panel) (*p<0.05; Dex *vs.* non-Dex-treatment, CTRL, bar = 50 µm). C. Satellite cells were cultured in differentiation media with Dex plus PBS or Dex plus myostatin inhibitor for 96 h, differentiated satellite cells were evaluated by immunostaining them with anti-eMyHC (green). The right panel summarizes the differentiation index (*p<0.05; Dex *vs.* non-Dex-treatment, CTRL, bar = 50 µm).

Regarding the mechanism for loss of muscle mass, we have found that chronic kidney disease (CKD) induces satellite cell dysfunction which contribute to loss of muscle mass [4]. To examine this mechanism in Dex-treated C57/BL6 mice, we isolated satellite cells and measured their functions (proliferation and differentiation) following Dex treatment. First, we confirmed there are glucocorticoid receptors (GR) in satellite cells by co-immunostaining the cells with anti-GR and anti-Pax7. We found that satellite cells from wild type mice express GR but that the GR was absent in satellite cells isolated from muscles of glucocorticoid receptor knockout mice (GRKO, Fig. 2A). We then incubated satellite cells isolated from muscles of WT mice with or without 10 µM Dex for 24 h and co-immunostained them with anti-Ki67 (a proliferation marker) and anti-Pax7 (a satellite cell marker): Dex significantly decreased Ki67 positive cells (Fig. 2B), suggesting that Dex suppressed satellite cell proliferation. Satellite cells incubated in differentiation media (DMEM plus 2% horse serum) with or without 10 µM Dex for 4 days were immunostained for eMyHC and as with proliferation, Dex-decreased satellite cell differentiation *vs.* results in non-Dex-treated satellite cells (Fig. 2C).

### Dex Suppresses Satellite Cell Function in vivo

**Figure 3:**
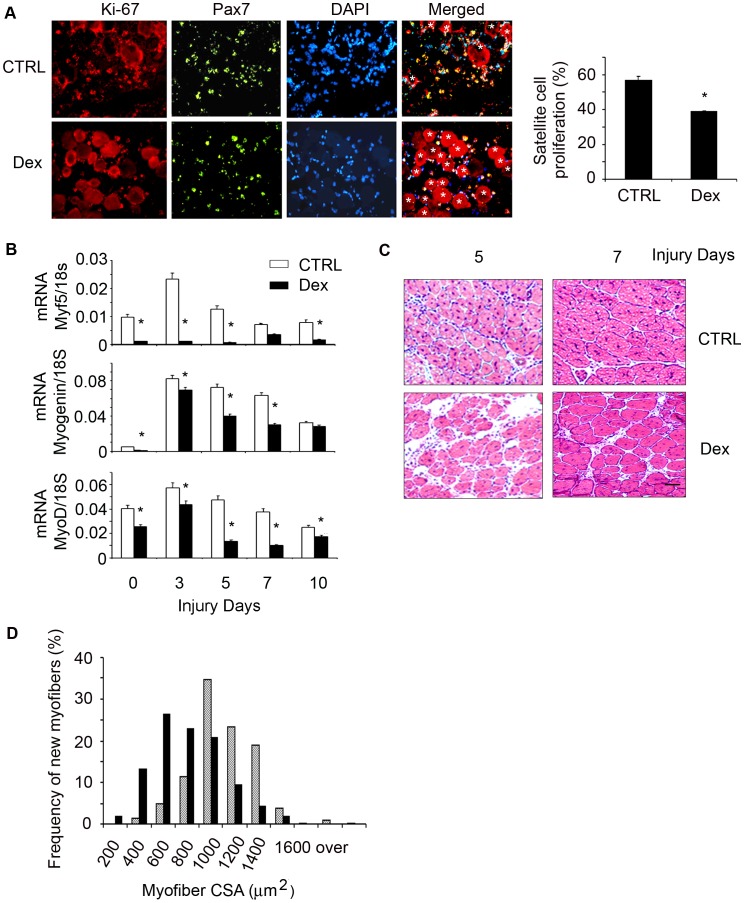
Dex suppresses satellite cell activation *in vivo*. C57/BL6 control mice were treated with Dex and TA muscles were injured. A. At 1.5 day following injury, the cryo-cross-section of injured muscle were co-immunostained with anti-Pax7 (green) and Ki-67 (red); in the merged (right) column, dual positive (proliferating) cells are indicated by yellow color. *indicates injured myofibers. The proliferation rate was shown in the right column. B. The mRNAs of Myf5, MyoD and Myogenin in non-injured (designated as 0 injury days) or injured muscles (at different time points) were assessed by RT-PCR (*p<0.05; Dex *vs.* non-Dex-treated CTRL mice; n = 3 mice for each group). C. H/E staining of the cross-section of injured muscles (bar = 50 µm). D. At 7 days after injury, the newly formed myofiber sizes were measured and the myofiber size distribution was presented.

To examine whether Dex causes satellite cell dysfunction *in vivo*, we used a standard model of muscle injury by CTX injection, because it activates satellite cells to proliferate, differentiate and develop into myofibers [32]. 12 week-old C57/BL6 mice were treated with Dex for 2 days before TA muscle were injured with CTX for 1.5 days. Cryo-cross-sections of injured TA muscles were co-immunostained with anti-Pax7 and Ki-67. We found significantly fewer Pax7 and Ki-67 dual positive cells in injured muscle of Dex treated mice *vs.* in injured muscle of control mice (Fig. 3A). At different time points following muscle injury, the mRNA expression of myogenic genes including Myf-5, MyoD and myogenin were significantly lower in muscles of Dex-treated mice *vs.* values in muscles of non-Dex-treated control mice (Fig. 3B). We also examined formation of new myofibers (indicated by myofibers with central nuclei) to evaluate whether the inhibition of myogenic genes by Dex was correlated with diminished satellite cell differentiation into muscle fibers. At 5 or 7 days after injury, muscle sections from Dex-treated mice had more space among newly generated myofibers and these new myofibers were smaller *vs.* results from injured muscle of control mice (Fig. 3C). At 7 days following injury, the size distribution of newly formed myofibers in muscles of Dex-treated mice was shifted to left (smaller myofiber direction) *vs.* the values in control mice (Fig. 3C). These data demonstrate that Dex causes loss of body and muscle weight in mice and suppresses satellite cell activation while impairing the regeneration of injured muscles (Fig. 1, 2&3).

### Dex Induces Myostatin Expression to Suppress Satellite Cell Activation

**Figure 4:**
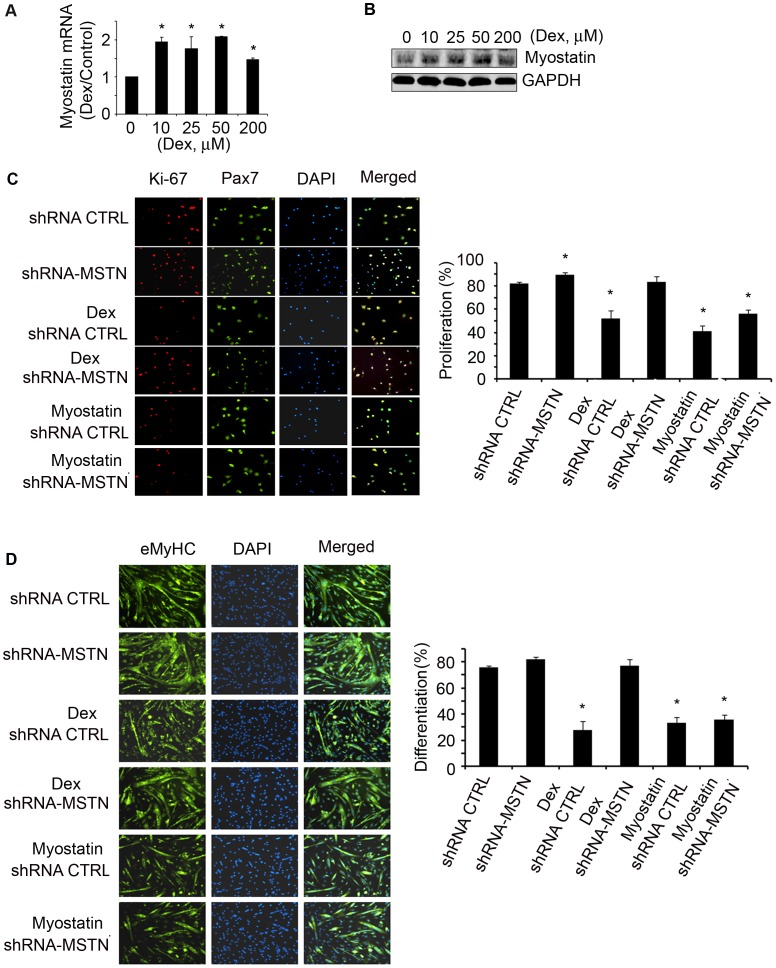
Dex stimulates myostatin expression in satellite cells suppressing their activation *in vitro*. A. satellite cells were treated with different concentrations of Dex for 24 h. Myostatin mRNA was evaluated by RT-PCR (*p<0.05 *vs.* non-Dex; n = 3 independent experiments). B. Satellite cells were treated with different concentrations of Dex for 36 h; a representative western blot of myostatin is shown. C. Satellite cells were transducted with shRNA-myostatin or shRNA-control lentivirus and treated with/without Dex or myostatin for 24 h. The percentage of Ki67 and Pax7 dual positive cells (indicated by yellow color in the merged column) to total Pax7 positive cells (green) is shown in right panel. (*p<0.05 *vs.* shRNA-control bar = 50 µm). D. Satellite cells were transducted with shRNA-myostatin or shRNA-control lentivirus then exposed to differentiation media with/without Dex or myostatin for 96 h. The fixed cells were immunostained with anti-eMyHC (left panel). The differentiation index is shown in the right panel (*p<0.05 *vs.* shRNA-control bar = 50 µm).

Because there are no glucocorticoid response elements in the promoter region of atrogenes or myogenic genes, GCs must cause loss of muscle weight by an indirect mechanism [9], [10]. GCs can induce myostatin expression, we examined whether Dex stimulation of myostatin suppresses satellite cell function. We examined this hypothesis in satellite cells isolated from muscles of WT mice and found that Dex did stimulate myostatin mRNA and protein expression in satellite cells (Fig. 4A&B). In addition, satellite cells treated with Dex had decreased proliferation (Fig. 2B) and inhibition of myostatin in Dex-treated satellite cells with the anti-myostatin peptibody (Myo-inh) significantly increased Ki67 expression *vs.* responses to Dex treatment only (Fig. 2B). Besides improving satellite cell proliferation, inhibition of myostatin also increased satellite cell differentiation despite Dex treatment (Fig. 2C). These results provide a potential mechanism for the catabolic effect of GC, Dex stimulates myostatin which suppresses satellite cell function. To further support this conclusion, we transducted isolated satellite cells with shRNA control or shRNA-myostatin lentivirus; myostatin knockdown was confirmed by western blot (data not shown). These cells were then treated with Dex for 24 h, and the fixed cells were co-immunostained with anti-Pax7 and Ki-67. The results indicate that knockdown of myostatin blocks Dex-suppressed satellite cell proliferation (Fig. 4C). Lentivirus transducted satellite cells were cultured with 2% horse serum to induce differentiation and treated with Dex for 96 h; knockdown of myostatin abolished Dex suppression of satellite cell differentiation (Fig. 4D). To confirm the specificity of myostatin knockdown with myostatin shRNA, we treated lentivirus transducted satellite cells with recombinant myostatin and found that myostatin reversed the effects of shRNA-myostatin on satellite cell proliferation and differentiation (Fig. 4C&D).

### Both Dex and Myostatin can Inhibit Akirin1 Expression in Satellite Cells

**Figure 5:**
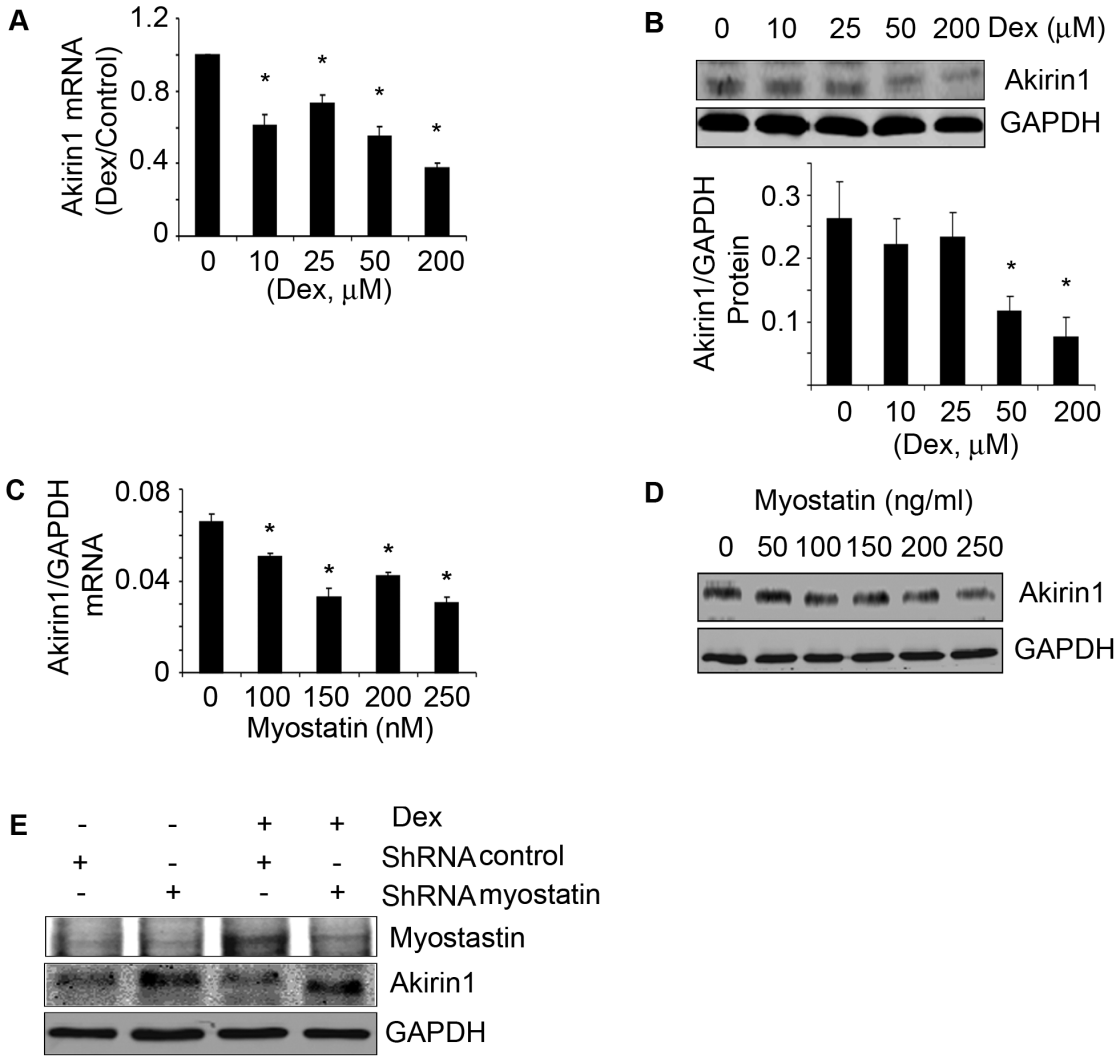
Dex or myostatin can inhibit Akirin1 expression. Satellite cells were treated with different concentrations of Dex for 24 h. A. Akirin-1 mRNA expression was examined by RT-PCR (corrected for GAPDH). The fold change *vs.* control (no Dex) are shown (*p<0.05; Dex *vs.* no-Dex; n = 3 independent experiments). B. A representative western blot of Akirin1 is in the upper panel. The band density of Akirin1 corrected for GAPDH are shown in lower panel. (*p<0.05; Dex vs. no-Dex; n = 3 independent experiments). C&D. Satellite cells were treated with different concentrations of myostatin. Akirin1 mRNA (C) and protein (D) was examined (*p<0.05 *vs.* no myostatin; n = 3 independent experiments). E. Stable cell lines were selected with puromycin from myoblasts transducted with lentivirus of shRNA-myostatin or shRNA-control and treated with or without Dex for 24 h. Representative Western blots of myostatin and Akirin1 are shown.

Although our results indicate that myostatin suppresses satellite cell proliferation and differentiation (Fig. 4), the mechanism underlying this response is not clear. Akirin1 is a target of myostatin and when satellite cells were treated with increasing concentrations of Dex, there was a dose-dependent increase in myostatin along with a decrease in both the mRNA and protein expression of Akrin1 (Fig. 5A&B, Fig. 4A&B) [17]. Moreover, when recombinant myostatin was added to satellite cells, Akirin1 mRNA and protein levels decreased in a dose-dependent manner (Fig. 5 C&D).

Next, we tested the hypothesis that the inhibition of Akirin1 by Dex is mediated by myostatin. Myostatin was knocked down in myoblasts using a shRNA-myostatin lentivirus. Control and myostatin knockdown cells were treated with 10 µM Dex for 24 h. In control cells, Dex increased myostatin and decreased Akirin1 protein expression (compare lane 1 with lane 3 in Fig. 5E). In contrast, in cells in which myostatin was knocked down, Dex treated cells had higher expression of Akirin1. Myostatin knockdown also significantly increased cell proliferation and differentiation even when Dex was present (Fig. 4C&D). The results indicate that Dex suppresses satellite cell activation by upregulating myostatin leading to inhibition of Akirin1 expression.

**Figure 6:**
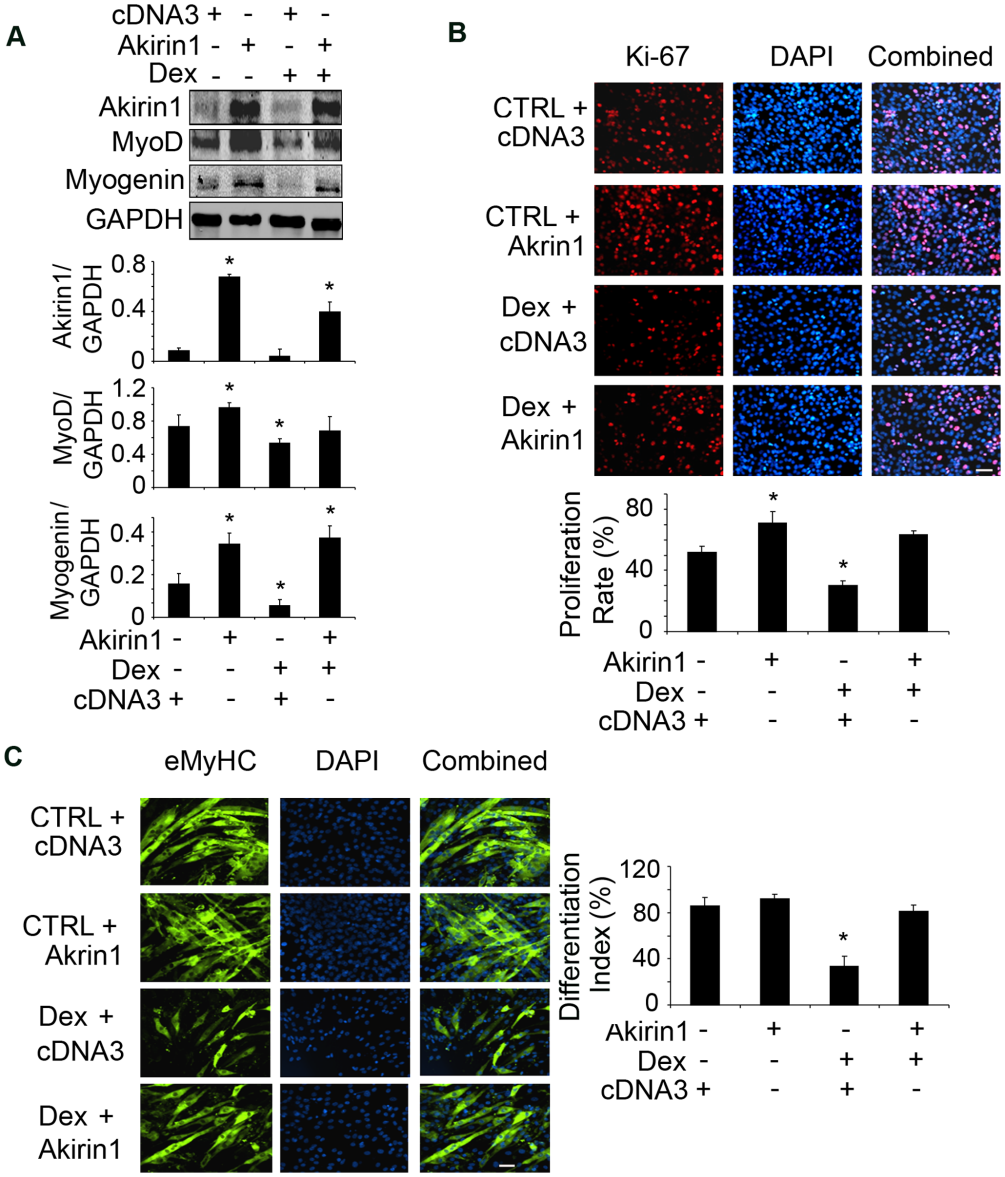
Overexpression of Akirin1 blocked Dex-induced suppression of myogenic gene expression and myoblast proliferation and differentiation. A. C2C12 myoblasts were transfected with Akirin-1 or cDNA3 (control). After 24 h, cells were treated with 10 µM Dex in 2% horse serum for 24 h. Representative western blots of measured proteins are shown (upper panel) and band density corrected for GAPDH is shown in lower panel. (*p<0.05 *vs.* cells transfected with cDNA3 without Dex treatment; n = 3 repeats). B. Transfected cells were treated with 10 µM Dex and immunostained with anti-Ki67 (red). The percentage of Ki67 positive cells to the total number of cells in 10 areas was examined (lower panel) (*p<0.05 *vs.* cells transfected with cDNA3 without Dex treatment). C. Transfected cells were incubated in 2% horse serum with or without 20 µM Dex for 96 h to stimulate differentiation. Cells were immunostained with anti-eMyHC (green, left panel). The differentiation index is shown in right panel (*p<0.05 *vs.* cells transfected with cDNA3 without Dex treatment; n = 3 repeats).

To document that Dex suppresses satellite cell function by inhibiting Akirin1 expression, we overexpressed Akirin1 in myoblasts; cDNA3 was transfected as control. Akirin1 expression increased the levels of MyoD and myogenin even when cells were treated with Dex (Fig. 6A). Increased Akirin1 expression also improved the proliferation and differentiation of myoblasts despite treatment with Dex (Fig. 6 B&C).

### Dex causes Loss of Muscle Mass and Satellite Cell Dysfunction via Myostatin in Mice

**Figure 7:**
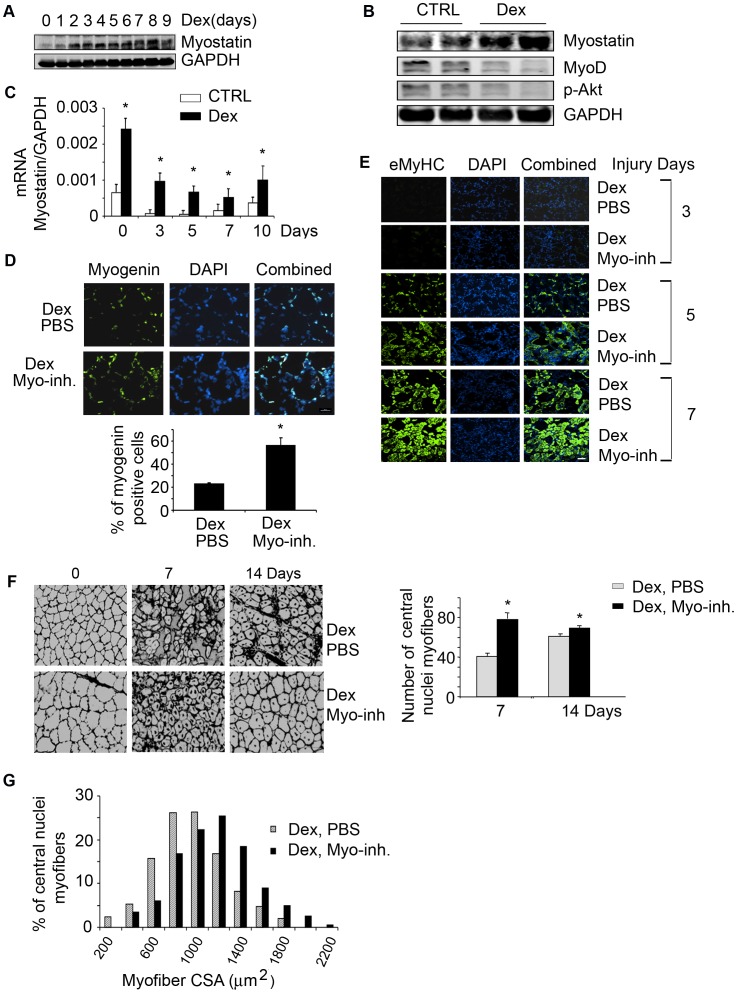
Dex increases myostatin expression and impairs satellite cell activation *in vivo*. A. Representative western blots of myostatin in gastrocnemius muscles of mice treated with Dex for different days. B. Representative western blots of indicated proteins from muscles of control or mice treated with Dex for 14 days. C. mRNA expression of myostatin was measured by RT-PCR in muscles of mice treated with or without Dex and injured with CTX (*p<0.05 *vs.* CTRL; n = 3 mice in each group). D. At 4 days after injury, cross-sections of muscle were immunostained with anti-myogenin (left panel) and the ratio of myogenin positive cells to DAPI expressed as a percentage is shown in right panel (*p<0.05 *vs.* Dex plus PBS). E. Cross-sections of injured TA muscles from mice injected with Dex plus PBS or Dex plus myostatin inhibitor were immunostained with anti-eMyHC (green). F. Sections in Fig. 7E were immunostained with laminin and DAPI to show the newly formed myofibers (left panel). The average number of central nuclei myofibers was calculated from 10 areas counted (*p<0.05 *vs.* Dex plus PBS, right panel). G. at 14 days after injury, newly formed myofiber cross-sectional areas were measured and the distribution is shown.

We tested our hypothesis *in vivo* by treating mice with Dex. From 2 to 9 days, myostatin expression increased in muscles of mice treated with Dex (Fig. 7A). At 14 days of Dex treatment, myostatin expression persisted and was associated with reduced levels of p-Akt and the satellite cell proliferation marker MyoD in muscles (Fig. 7B). To determine if Dex impairs the abilities of satellite cells to repair injured muscle, we treated mice with Dex for 5 days and then injected CTX to induce TA muscle damage. Unexpectedly, injury decreased myostatin mRNA but was still significantly higher in muscles of Dex-treated mice *vs.* non-Dex-treated mice in both injured and non-injured muscles (Fig. 7C).

Since myostatin negatively influenced satellite cell functions in cultured cells (Fig. 4), we tested the hypothesis that inhibition of myostatin in Dex-treated mice would increase satellite cell activity and improve muscle regeneration after injury. We injected anti-myostatin peptibody (6.25 mg/kg) every other day into mice because this dose can effectively block myostatin expression and block CKD-induced muscle wasting [20]. Mice received Dex plus PBS or Dex plus the anti-myostatin peptibody and TA muscles were injured for 4 days, cross-sections of injured muscles were immunostained with the satellite cell myogenic marker, anti-myogenin; number of myogenin positive cells was significantly higher in injured muscles of mice treated with Dex plus the myostatin inhibitor *vs.* values in mice that were treated with Dex plus PBS (Fig. 7D). Myogenin positive cells were not found in non-injured muscles (data not shown). We also evaluated satellite cell differentiation plus formation of new myofibers by immunostaining cross sections of injured TA muscles with eMyHC. At 3 days after injury, no new myofibers were present in mice treated with Dex plus the myostatin inhibitor or with Dex plus PBS. After 5 days of injury, the inhibition of myostatin in Dex-treated mice resulted in more newly formed myofibers (eMyHC positive cells) *vs.* results in mice treated with Dex plus PBS; this response was more pronounced at day 7 after injury (Fig. 7E). Sections of muscle immunostained with laminin and DAPI at 7 days after injury found increased numbers and sizes of myofibers with central nuclei in mice treated with Dex plus the anti-myostatin peptibody (Fig. 7F). At 14 days after injury, the distribution of myofiber sizes was shifted to the right (larger) in Dex plus anti-myostatin peptibody-treated mice *vs.* Dex-PBS-treated mice (Fig. 7G). Inhibition of myostatin in Dex-treated mice also led to increased body and muscle weights and myofiber sizes (Fig. S1 A, B&C). The increase in body and muscle weight was accompanied by a decrease in the expression of the E3 ubiquitin ligases Atrogin-1 and MuRF-1 (Fig. S1D). Taken together, the results suggest that blocking myostatin *in vivo* abrogates the inhibited satellite cell activation that is induced by Dex. The result is improved muscle regeneration and growth.

### In Activated Satellite Cells Dex Decreases Akirin1 Expression by a Myostatin Pathway

**Figure 8:**
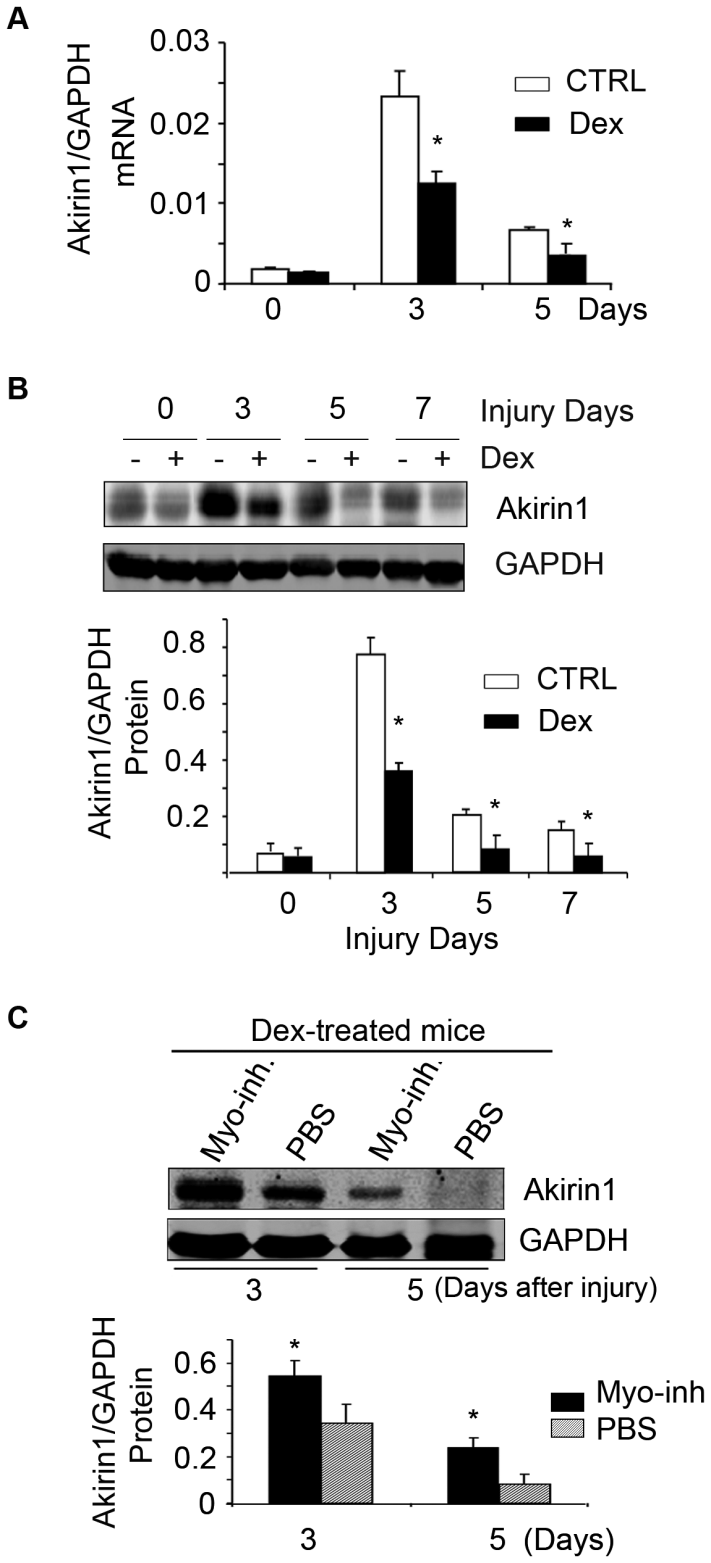
Dex decreases Akirin1 expression in activated satellite cells via myostatin expression in mice. **A**. Akirin1 mRNA was evaluated in injured muscle of mice treated with/without Dex (*p<0.05 *vs.* no-Dex; n = 5 mice). B. Representative western blot of Akirin1 from injured muscle of mice treated with/without Dex (upper panel). The density of Akirin1 corrected for GAPDH is in lower panel (*p<0.05 *vs.* ctrl; n = 5 mice). C. A representative western blot of Akirin1 from injured TA muscles of mice treated with Dex plus PBS or Dex plus myostatin inhibitor (upper panel). The density of Akirin1 corrected for GAPDH is in lower panel (*p<0.05 *vs.* Dex plus PBS; n = 5 mice).

We evaluated Akirin1 expression in muscles of mice that had been treated with Dex. In injured muscle of non-Dex-treated, control mice, both the mRNA and protein expressions of Akirin1 increased while in injured muscles of Dex-treated mice, Akirin1 expression was significantly lower *vs.* values in non-Dex-treated mice (Fig. 8 A&B). Finally, we injected PBS or the myostatin inhibitor into Dex-treated mice with injured TA muscles. In injured muscles of mice treated with Dex plus the myostatin inhibitor, Akirin1 levels were significantly higher *vs.* values in mice treated with Dex plus PBS (Fig. 8 C) suggesting that Dex decreases Akirin1 in satellite cells by a myostatin-mediated mechanism.

## Discussion

There have been reports exploring how GCs cause muscle wasting. First, GCs stimulate protein degradation in the ubiquitin proteasome systems by leading to an increase in the expression of muscle-specific E3 ubiquitin ligases, Atrogin-1/MAFbx and MuRF-1. In this case, however, the expression of atrophy-related genes are not directly regulated by GCs because there are no glucocorticoid response element binding sites in the promoter region of atrogenes [7], [33]. Second, physiologic levels of GCs were shown to stimulate an interaction between the activated GC receptor and p85 of PI3K/Akt in muscle. The interaction stimulates protein breakdown in muscle by inhibiting Akt phosphorylation [22]. In the present studies, we uncovered a third mechanism, namely that GCs impair satellite cell functions in muscle. The mechanism for this response is that GCs suppress satellite cell activation by inhibiting the expression of Akirin1, a gene associated with satellite cell activation. GC induces inhibition of Akirin1 by stimulating myostatin. Specifically, we have shown that GCs stimulate myostatin expression in isolated satellite cells and in muscles of mice. But, when myostatin was inhibited by anti-myostatin peptibody or by treating myoblast with shRNA-myostatin, the response to GCs that inhibits Akrin1 expression is blocked. We also show that an increase in Akrin1 potentiates myoblast proliferation and differentiation when GCs are present. Thus, GCs suppress the ability of satellite cell to counteract muscle growth.

How do GCs affect satellite cell function? Dex suppresses satellite cell proliferation and differentiation both *in vitro* (Fig. 2) and in muscles of satellite cells activated by injury (Fig. 3). The signaling pathway by which an increase in GCs causes satellite cell dysfunction involves an increase in myostatin expression. The myostatin activation can be linked to putative glucocorticoid response elements (GREs) in the myostatin promoter and our finding that Dex up-regulates myostatin mRNA and its protein in cultured satellite cells (Fig. 4) [34], [35]. An increase in myostatin is relevant because it is expressed in satellite cells and reportedly regulate satellite cell quiescence and self-renewal [14]. Indeed, we found that in mice treated with Dex, there was increased myostatin expression in muscle associated with satellite cell dysfunction (Fig. 7 A&B). Proof of the myostatin role was obtained by inhibiting it. When myostatin was blocked, satellite cell functions improved and the number of activated satellite cells in injured muscles increased (Fig. 7D). Thus, GC induces myostatin expression in satellite cells leading to inhibition of satellite cell activity (Fig. 7).

Besides confirming that myostatin adversely affects satellite cell activity [14], [36]–[38], we uncovered another major mediator of satellite cell response to GC stimulation. Specifically, we found that Akirin1 counteracts the GC-induced suppression of satellite cells; it improves the functions of satellite cells including an increase in the expression of MyoD and myogenin (Fig. 6). The changes in satellite cell function stimulated by Akirin1 are complicated because Akirin1is a target of myostatin. For example, the level of Akirin1 increases in muscles of myostatin KO mice and it is also increased in activated satellite cells and in injured muscles (Fig. 8) [17], [39]. We probed these results further by treating satellite cells with recombinant myostatin; there was a decrease in Akirin1 mRNA and expression of its protein (Fig. 5C&D). These results explain how inhibition of myostatin led to an increase in Akirin1 expression in muscles of mice or in satellite cells leading to improved muscle regeneration (Fig. 7) and muscle growth (Fig. S1). In addition, we showed that knock down of myostatin increased Akirin1 expression and enhanced myoblast or satellite cell proliferation and differentiation even in the presence of Dex (Fig. 5 E, 4 C&D). Contrariwise, over-expression of Akirin1 in muscle cells increased the expression of myogenic genes, MyoD and myogenin even when Dex was present (Fig. 6). The results suggest that Akirin1 functions upstream of MyoD. This is relevant because MyoD, like Twist, is a basic Helix-loop-Helix (bHLH) transcription factor and reportedly, Akirin1 interacts with Twist in a chromatin remodeling complex which promotes gene expression during embryogenesis [19]. Combining these results, we conclude that Akirin1 regulates MyoD to influence satellite cell function and we plan to explore this proposal in future studies.

Regarding to responses to injury, we found that myostatin expression decreased in injured muscles of control mice while the expression of satellite cell myogenic genes increased (Fig. 7C). These results are consistent with those of Cornelison et al, who concluded that myostatin was significantly down-regulated in activated satellite cells; they suggest that an increase in myostatin could be involved in maintaining satellite cell quiescent [40]. In agreement with that conclusion, we found that Dex increased myostatin expression in both injured and non-injured muscles (Fig. 7A&C) and that inhibition of myostatin stimulated satellite cell function to prevent Dex-induced loss of body and muscle weight (Fig. S1 & Fig. 7E&F).

Inflammation and myofiber necrosis occurs after muscle injury and these responses are associated with secretion of cytokines and growth factors that can influence satellite cell activation [41]. In our results, Dex decreased mRNA levels of several cytokines and chemokines in injured muscles (Fig. S2). Potentially, decreases in the expression of genes could contribute to inadequate muscle regeneration responses in Dex-treated mice. Indeed, Salerno et al reported that Akirin1 is expressed in macrophages and participates in chemotaxis of both macrophages and myoblasts into injured muscle [39]. Our finding that Dex suppresses Akirin1 expression in satellite cells as well as injured muscle could extend the proposal about changes in chemotoxins; specifically reduction in Akirin1 could reduce macrophage infiltration into injured muscles, thereby reducing the release of cytokines, chemokines and growth factors. These are prominent features of muscle repair and could impair or suppress satellite cell activation. Dex affects these factors will be explored in the future.

In summary, we have defined a new mechanism for GC-induced loss of muscle mass. The results add insights into the regulation of satellite cell function. Specifically, GCs suppress satellite cell function by upregulating myostatin and inhibiting Akirin1. Together these responses suppress MyoD expression and the participation of satellite cells in the process of muscle repair.

## Supporting Information

Figure S1 **Myostatin inhibition prevents Dex induced body and muscle weight loss**. Mice were treated with Dex plus PBS or Dex plus anti-myostatin peptibody for 14 days. A. Body weight changes (*p<0.05 *vs.* CTRL mice; n = 8 mice). B. Gastrocnemius muscle weight (*p<0.05 *vs.* CTRL mice; n = 8 mice). C. Myofiber distribution. D. Representative western blots of Atrogin-1 or MuRF-1.
(TIF)

Figure S2 **Dex inhibits expression of inflammatory genes in injured muscle**. Mice were treated with Dex for 14 days and TA muscles were injured at different times. mRNA expression of inflammatory genes was evaluated by RT-PCR. Primer sequences will be sent upon request.
(TIF)
