# Supplementary figures and images for: Myostatin Suppression of Akirin1 Mediates Glucocorticoid-Induced Satellite Cell Dysfunction
Source: PLoS One. 2013 Mar 13;8(3):e58554. doi: 10.1371/journal.pone.0058554 (PMC3596298; doi:10.1371/journal.pone.0058554)

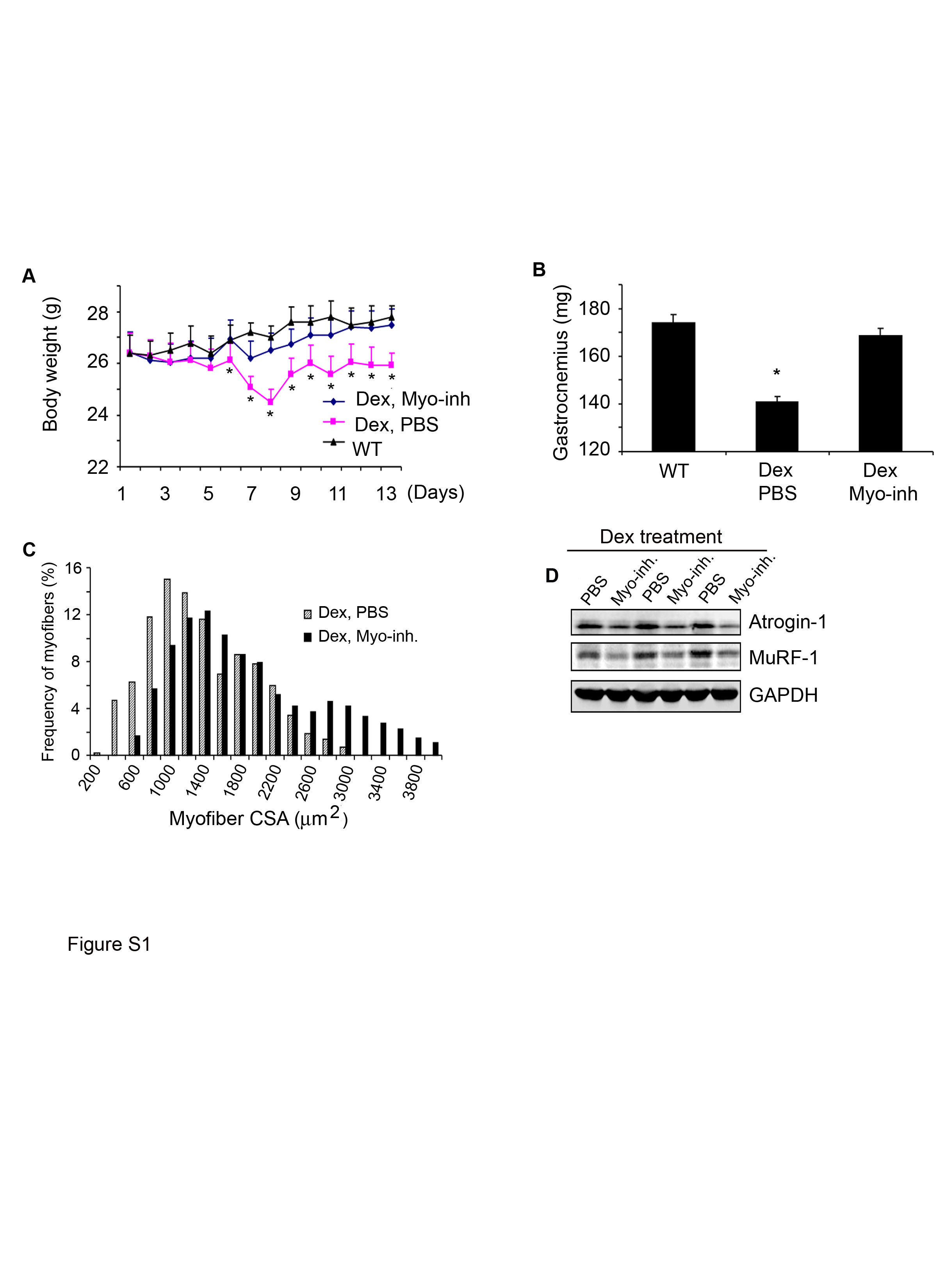

Supplement: Figure S1 — Myostatin inhibition prevents Dex induced body and muscle weight loss. Mice were treated with Dex plus PBS or Dex plus anti-myostatin peptibody for 14 days. A. Body weight changes (*p<0.05 vs. CTRL mice; n = 8 mice). B. Gastrocnemius muscle weight (*p<0.05 vs. CTRL mice; n = 8 mice). C. Myofiber distribution. D. Representative western blots of Atrogin-1 or MuRF-1. (TIF) [file pone.0058554.s001.tif]

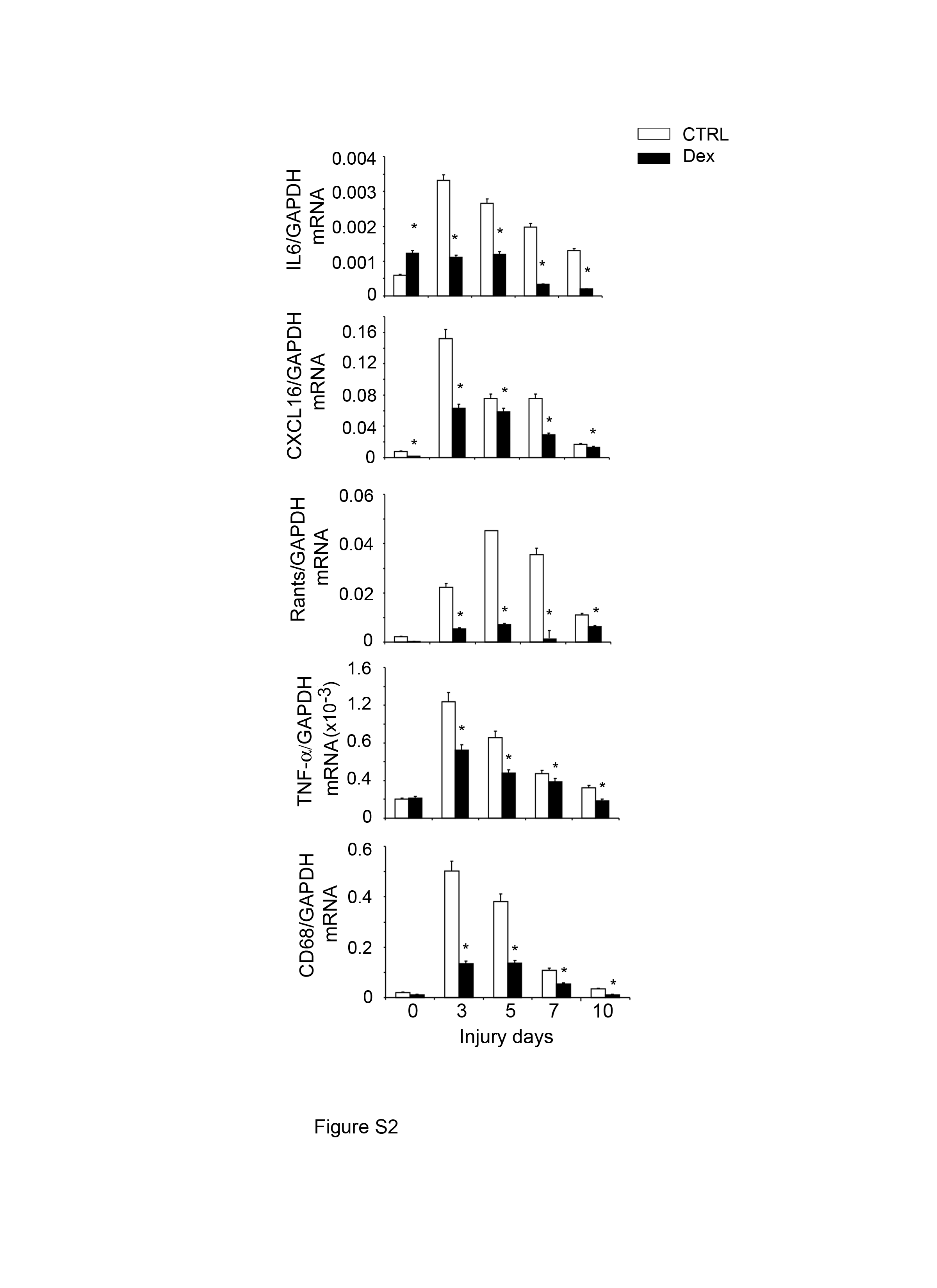

Supplement: Figure S2 — Dex inhibits expression of inflammatory genes in injured muscle. Mice were treated with Dex for 14 days and TA muscles were injured at different times. mRNA expression of inflammatory genes was evaluated by RT-PCR. Primer sequences will be sent upon request. (TIF) [file pone.0058554.s002.tif]
